# Non-Destructive Detection of Pomegranate Blackheart Disease via Near-Infrared Spectroscopy and Soft X-ray Imaging Systems

**DOI:** 10.3390/foods14142454

**Published:** 2025-07-12

**Authors:** Rongke Nie, Xingyi Huang, Xiaoyu Tian, Shanshan Yu, Chunxia Dai, Xiaorui Zhang, Qin Fang

**Affiliations:** 1 School of Food and Biological Engineering, Jiangsu University, Xuefu Road 301, Zhenjiang 212013, China; nierongke@126.com (R.N.); tianxy@ujs.edu.cn (X.T.); yushannie_lucky@163.com (S.Y.); 15751003723@163.com (X.Z.); fangqin200011@163.com (Q.F.); 2 School of Electrical and Information Engineering, Jiangsu University, Xuefu Road 301, Zhenjiang 212013, China; txdcx@126.com

**Keywords:** pomegranate, food non-destructive detection, blackheart disease, near-infrared spectroscopy, soft X-ray

## Abstract

Pomegranate blackheart disease, as an internal disease affecting the global pomegranate industry, is difficult to identify externally and urgently requires non-destructive detection methods for rapid diagnosis. This study established discriminative models for blackheart disease severity in pomegranates by using near-infrared (NIR) spectroscopy and soft X-ray imaging techniques. The results showed that the optimal NIR-based discriminative model, constructed with a Random Forest (RF) algorithm based on spectra preprocessed by the second-derivative (D2) denoising and a Competitive Adaptive Reweighted Sampling (CARS) algorithm, achieved a prediction set accuracy of 86.00%; the optimal soft X-ray imaging-based discriminative model, built with an RF algorithm using textural features extracted from images preprocessed by median filtering and a Contrast-Limited Adaptive Histogram Equalization (CLAHE) algorithm combined with gray-level co-occurrence matrix (GLCM) and gray-gradient co-occurrence matrix (GGCM) algorithms, reached a prediction set accuracy of 93.10%. In terms of model performance, the model based on soft X-ray imaging exhibited superior performance. Both techniques possess distinct advantages and limitations yet enable non-destructive detection of pomegranate blackheart disease. Further technical optimizations in the future could provide enhanced support for the healthy development of the pomegranate industry.

## 1. Introduction

Pomegranate, indigenous to Central Asia (notably Iran and Afghanistan), is rich in diverse vitamins and polyphenols. Its sweet–tart flavor profile and documented dermatological benefits contribute to its broad consumer appeal. The fruit is extensively consumed fresh and processed into multiple value-added forms—including juices, jams, jellies, vinegar, wines, seed oils, and nutraceutical extracts—which enjoy substantial market acceptance globally.

However, pomegranates are susceptible to contamination by pathogenic fungi during harvesting and transportation, which can result in reduced fruit quality and yield. Pomegranate blackheart disease [1,2], also referred to as heart rot, is a major pathogen-induced disease that significantly affects pomegranate production worldwide. This internal disease is caused by infection with Alternaria species, which degrade the fruit’s cuticle and cell walls, triggering a series of physiological changes in the host tissue [3]. Upon opening the infected fruit, brown (soft) to black (dry) rot of the arils is observed. Notably, the external, hard skin of the fruit often appears healthy, masking the internal decay [4]. Due to the internal rot, infected fruit produces a hollow sound when struck, in contrast to the dull sound emitted by healthy fruit.

Currently, no effective control measures exist to prevent the spread of this disease, and healthy pomegranates can also become infected during storage, leading to significant losses. Once infected fruit enters the market, it not only negatively impacts the pomegranate industry but also poses food safety risks. Consequently, consumers may reject the affected pomegranates, resulting in a damaged brand reputation [5]. This rejection can adversely affect the long-term sales of pomegranate suppliers and cause substantial financial losses for pomegranate growers. According to reports, the losses of pomegranate products caused by Alternaria species could be substantial, with over 50% of the fruits in some areas being infected by blackheart disease [2].

However, because blackheart disease primarily affects the internal tissue, infected fruit often lacks external visual symptoms, making it challenging to identify at early stages. Presently, detection of pomegranate blackheart disease is largely reliant on expert analysis, where specialists use their experience to differentiate between healthy and infected fruit [6]. This method is highly subjective and prone to false positives or negatives, in addition to being time-consuming and labor-intensive. Therefore, there is a pressing need for the development of rapid, non-destructive detection technologies capable of assessing the internal condition of pomegranates and identifying infection [7].

Zhang et al. [8] employed nuclear magnetic resonance imaging (NMR) technology to analyze magnetic resonance (MR) images of both infected and healthy pomegranate fruits. Their study revealed that the edge of the false seed coat in the lesion area appeared blurred, whereas the healthy false seed coat was clearly delineated. Using the signal intensities in the image, the partial least squares discriminant analysis (PLS-DA) model was able to correctly identify the presence of black heart in pomegranate fruit with an accuracy of over 92%. Arendse et al. [9] employed X-ray computed tomography (CT) coupled with high-density reference phantoms, and the researchers established a non-destructive classification framework for pomegranate black heart disease. Crucially, the study revealed an inverse density–pathology correlation where disease severity escalates with diminishing tissue density. However, both aforementioned studies are constrained by suboptimal detection throughput, fundamentally compromising their translational viability for industrial implementation. In a similar vein, Myresiotis et al. [10] established a rapid detection method for Alternaria toxins in pomegranate samples, utilizing a quick, easy, cheap, effective, rugged, and safe method (QuEChERS) approach which yielded favorable results. The overall average recoveries ranged from 82.0% to 109.4% and the relative standard deviations were from 1.2% to 10.9%. However, this study mandates the implementation of destructive methodology to achieve its objectives. Munera et al. [11] established a blackheart disease detection model for pomegranates by integrating X-ray imaging with image processing algorithms, achieving a classification accuracy of 93.3%. Nevertheless, their sample acquisition methodology and image processing pipeline require further refinement. Therefore, to better serve industrial inspection settings, it is essential to achieve rapid, non-destructive detection of pomegranate blackheart disease.

The principle of NIR spectroscopy originates from the selective absorption of specific NIR wavelengths by hydrogen-containing groups in substances. This non-destructive analytical method combines NIR spectrometers, chemometrics, and mathematical modeling, with notable applications in detecting internal defects in fruits for agricultural quality assurance [12]. NIR spectroscopy has been successfully applied to apple moldy core disease detection [13], online non-destructive identification of internal mold infestation in sand pears [14], fungal contamination monitoring in walnut kernels [15], and pest infestation detection within fruits [16], providing effective solutions for internal disease and quality assessment. Furthermore, NIR spectroscopy demonstrates extensive applications in quantitative analysis, including in moisture content determination in black garlic and bread [17,18], soluble solids content (SSC) measurement in apples [19], total acidity assessment in Shanxi aged vinegar [20], and pH value detection in cocoa beans [21].

Soft X-ray imaging utilizes differential X-ray attenuation across materials of varying densities. The penetrating radiation intensity attenuates proportionally to encountered density gradients, with detectors converting transmitted signals into digital data. Computational analysis of these signals’ spatial distribution enables the reconstruction of internal density variations. This non-destructive technique allows for effective visualization and quantitative assessment of internal defects in fruits and agricultural products [22]. Matsui et al. [23] developed an automated detection model for stem-end rot in *Hass* avocado fruits using soft X-ray imaging technology. Van et al. [24] achieved automated segmentation of internal healthy and diseased tissues in citrus fruits through soft X-ray image acquisition. Francisca et al. [25] employed soft X-ray imaging combined with a Convolutional Neural Network (CNN) to precisely localize the position of seed decay within mango fruits.

Although NIR spectroscopy and soft X-ray imaging technology have demonstrated effective applications in internal quality inspections of other fruits, their utilization in detecting pomegranate blackheart disease has not yet been identified. As two rapid and non-destructive detection methods, the successful implementation of these technologies for pomegranate blackheart disease detection would significantly reduce costs and enhance efficiency compared to conventional detection approaches. Therefore, this study employed NIR spectroscopy and soft X-ray imaging technology combined with machine learning algorithms to establish detection models for pomegranate blackheart disease and systematically evaluated the performance of the models.

## 2. Materials and Methods

**Figure 1:**
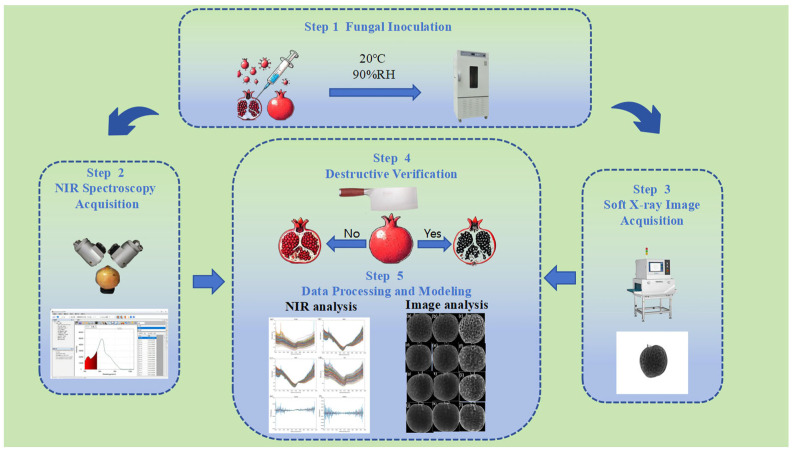
Flowchart of the pomegranate blackheart disease detection experiment.

Figure 1 illustrates the experimental workflow. It comprised the following five sequential phases: (1) preparation of disease-infected pomegranate samples by artificial inoculation with pathogenic fungi; (2) acquisition of transmittance spectra from internal pomegranate tissues; (3) acquisition of internal soft X-ray images of pomegranate samples; (4) destructive verification of disease severity in pomegranate samples; (5) parallel processing of spectral datasets and X-ray image features followed by the construction of independent discriminant models.

### 2.1. Samples Preparation

In September 2024, 192 healthy pomegranates of similar sizes and free from pests and diseases were purchased from a pomegranate orchard in Mengzi, Yunnan. The fruits were transported to the laboratory via cold-chain logistics and stored at 25 °C for 24 h to equilibrate to ambient temperature. Compared with healthy samples, diseased samples present significantly higher acquisition challenges due to the impracticality of obtaining substantial diseased samples through manual harvesting. Reliance on destructive sampling methods for diseased samples collection would eliminate the possibility of subsequent non-destructive testing, whereas the application of high-cost imaging techniques (e.g., magnetic resonance imaging, computed tomography) for sample acquisition results in low acquisition efficiency and constrained sample quantities per session. Therefore, standardized experimental protocols for artificial disease induction must be established to systematically generate pathological sample data. Among these, 96 pomegranates were inoculated with fungal suspensions as the infected group, while the remaining 96 served as the healthy control group (without fungal inoculation). The experimental procedures are detailed in the follow paragraphs.

#### 2.1.1. Fungal Cultivation

**Figure 2:**
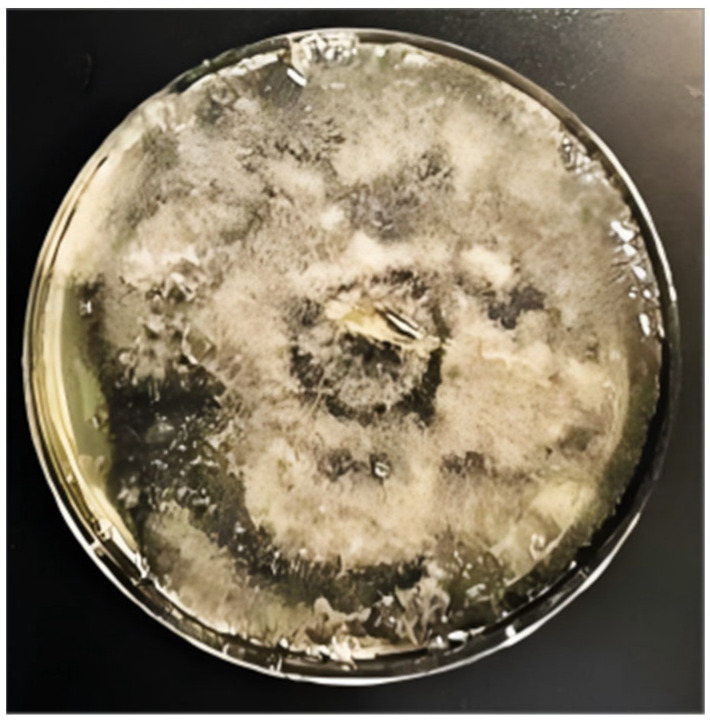
Cultivated *Alternaria alternata*.

Under aseptic conditions, the *Alternaria alternata* slant culture was opened, and fungal colonies were extracted using an inoculating hoe. The colonies were fragmented with an inoculating shovel and transferred to Potato Dextrose Agar (PDA) plates. The plates were sealed for one week until the fungal colonies fully covered the medium surface (as shown in Figure 2).

#### 2.1.2. Fungal Inoculation

*Alternaria alternata* mycelia were transferred to a pre-prepared 10 mL sterile saline solution in a centrifuge tube. The tube was tightly sealed and vortexed at high speed for 10 min to homogenize the suspension. The spore concentration was quantified using a hemocytometer and adjusted to 3 × 10^5^ CFU/mL for subsequent injection. Prior to injection, the pomegranate surfaces were sterilized with 5% sodium hypochlorite solution to prevent microbial contamination. Fungal inoculation was performed by injecting the suspension into the calyx region using a sterile syringe. Single injections were administered using 1 mL disposable syringes with a dosage of 0.3 mL per injection. To ensure experimental reliability, syringes were strictly prohibited from reuse to prevent cross-contamination.

**Figure 3:**
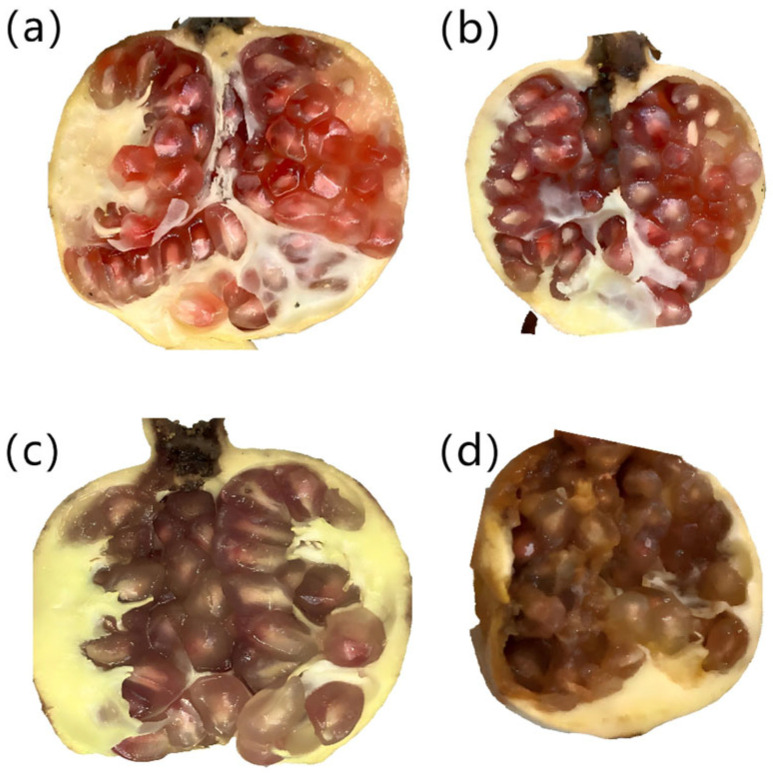
Cross-sectional views of pomegranates at different days after infection with blackheart disease. (**a**) Healthy; (**b**) 1 day after infection; (**c**) 3 days after infection; (**d**) 5 days after infection.

Based on Ezra’s research [26], *Alternaria alternata* exhibits accelerated growth under 20 °C and high-humidity conditions, reaching peak activity around the sixth day. Therefore, the disease development process was conducted in a constant temperature and humidity chamber maintained at 20 °C and 90% RH. Preliminary experiments revealed that pomegranates inoculated with the fungus developed near-complete blackheart disease decay by the fifth day under optimal conditions (Figure 3). To align with practical diagnostic needs focusing on early- and mid-stage disease identification, the 96 infected pomegranates were further subdivided into three groups. To enable simultaneous detection experiments on pomegranates with varying disease severity levels at the same time point, three groups of pomegranates were inoculated with fungi at different time intervals. Among these, the group inoculated first developed the longest disease progression period and most severe symptoms, whereas the group inoculated last exhibited the shortest disease progression period and mildest symptom development. The grouping of pomegranate samples is listed in Table 1.

### 2.2. NIR Spectroscopy Acquisition

**Figure 4:**
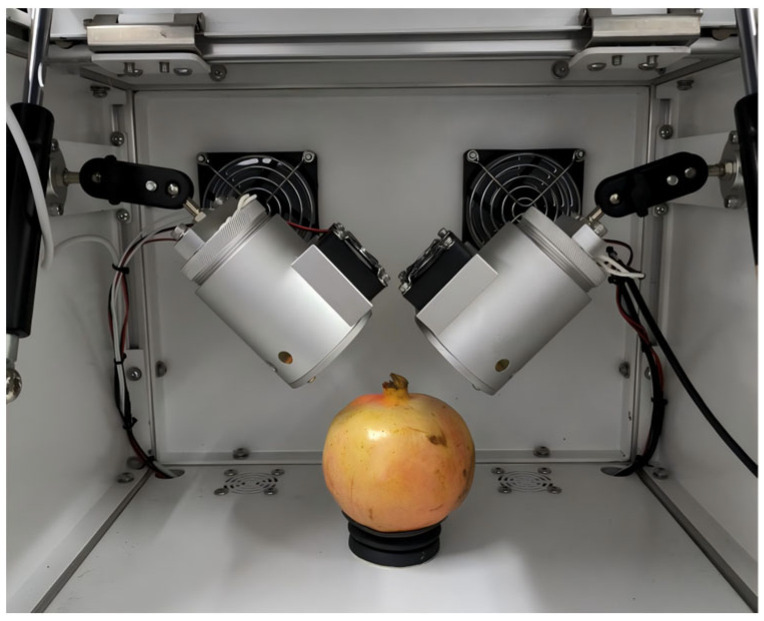
Spectral acquisition system.

The custom spectral acquisition system developed at the authors’ institution is illustrated in Figure 4. It consists of two symmetrically arranged halogen lamps with cooling fans, and a food-grade silicone tray fixed at the center to secure fruit positioning and provide light shielding. The entire setup is enclosed within an aluminum alloy chamber to prevent interference from ambient light. The SE2050 spectrometer (OtO Photonics Inc., Taiwan, China) and other detection components are housed at the base, while a collimating lens positioned at the center of the silicone tray interfaces with the spectrometer to capture transmitted spectral data through the pomegranates.

The light sources were positioned 20 cm above the baseplate at a 45° angle relative to the vertical normal. Instrumental parameters were configured as follows: integration time of 500 ms, scan averaging count of 3, and smoothing width of 5. The spectrometer was connected to a computer via a USB cable, enabling real-time transmission of acquired spectral data to the computer system.

### 2.3. Soft X-Ray Image Acquisition

**Figure 5:**
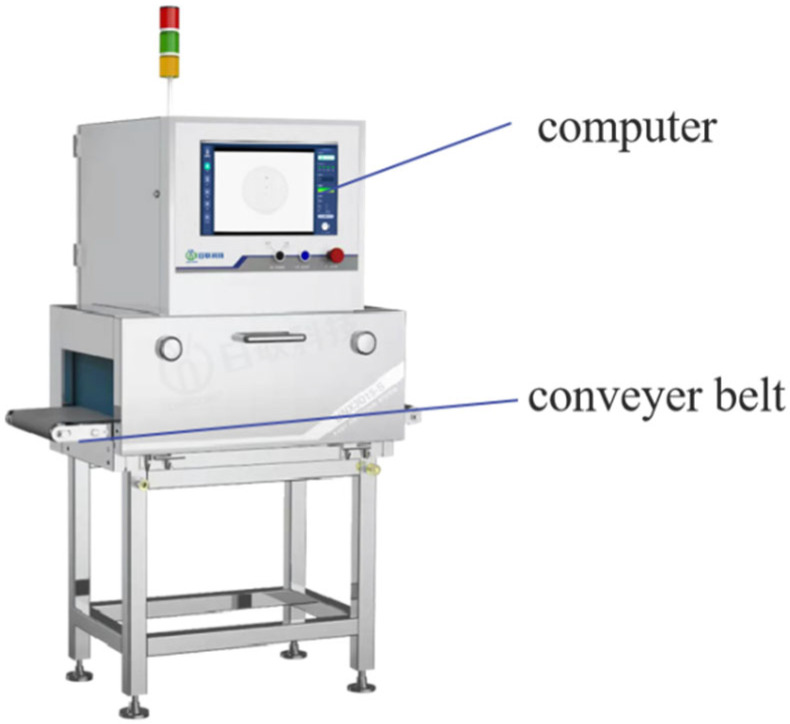
Soft X-ray imaging device.

Following spectral data acquisition, all pomegranate samples were securely packaged in anti-collision soft foam and immediately transported to Wuxi UNICOMP Technology Co., Ltd. (Wuxi, China). Imaging was performed using a UNX3015-S system (as shown in Figure 5), which integrates a conveyor belt and an X-ray imaging assembly. Operational parameters were set as follows: tube voltage at 80 kV, tube current at 2 mA, and gamma value at 1. To prevent the rotational movement of pomegranates during conveyor belt transport, a low-density polyethylene foam board (6 cm thick) was affixed to the belt, featuring an 8 cm diameter central aperture to securely embed each fruit. Preliminary trials established a maximum conveyor speed of 0.4 m/s, as higher velocities risked sample rotation and compromised image quality. After parameter optimization, sequential imaging was conducted for all samples.

### 2.4. Destructive Verification

After completing non-destructive testing (NDT) on pomegranate samples, the specimens were cut along the calyx and cross-sectional images were captured vertically using an industrial camera. The internal seed regions of the pomegranate were segmented, and based on expert evaluation, the blackheart disease areas within the images were identified. The pixel ratio of the diseased regions to the total cross-sectional area of the pomegranate was calculated to quantitatively characterize the actual infection level. The disease severity obtained through destructive testing was recorded and served as the dataset foundation for subsequent discriminant model development.

### 2.5. Preprocessing of NIR Spectroscopy

To enhance the quality of spectral data, the Local Outlier Factor (LOF) algorithm and the Mahalanobis distance method were employed to eliminate outliers from sample spectra [27,28,29]. A Partial Least Squares Discriminant Analysis (PLS-DA) model was established on the remaining dataset after outlier elimination [30,31,32]. The effectiveness of outlier removal was evaluated using metrics including testing set accuracy, F1-score, five-fold average accuracy, and five-fold average F1-score.

During practical NIR spectroscopic measurements, factors including particle size inhomogeneity, surface texture variations in samples, and minor environmental fluctuations during measurement may introduce noise interference, compromising both spectral data quality and the accuracy of subsequent analytical results. These interferences pose significant challenges to subsequent data analysis and feature extraction processes. To mitigate adverse effects induced by noise, five distinct denoising methodologies were implemented on spectral data after outlier removal. The specific methods used are as follows: Multiplicative Scatter Correction (MSC), Standard Normal Variate (SNV) transformation, Savitzky–Golay (SG) smoothing algorithm, and derivative algorithms (first derivative and second derivative) [33]. Based on the outlier-removed spectral data and different denoising processing algorithms, classification models for pomegranate blackheart disease were established by integrating Random Forest (RF) and Support Vector Machine (SVM) algorithms [34].

To further optimize the model’s performance, three algorithms—Competitive Adaptive Reweighted Sampling (CARS), Successive Projections Algorithm (SPA), and Principal Component Analysis (PCA)—were employed to enhance the model’s performance [35,36,37,38]. The model performance was evaluated using accuracy rates, F1-scores, and confusion matrices from both training and prediction sets [39]. The entire preprocessing and modeling procedures were implemented using MATLAB 2023b (MathWorks, Natick, MA, USA) software.

### 2.6. Preprocessing of Soft X-Ray Image

**Figure 6:**
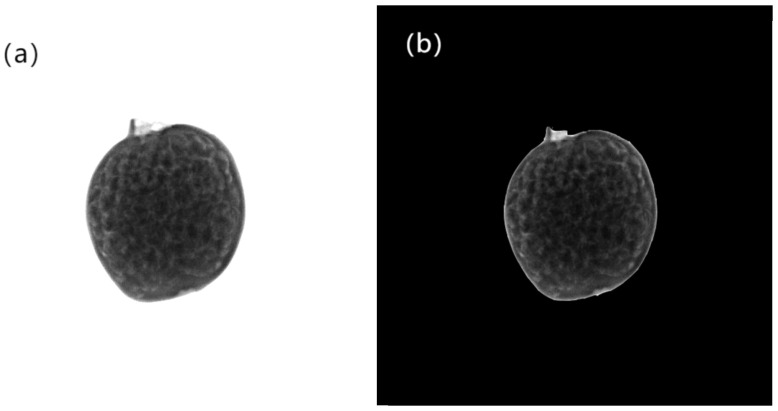
The segmentation process of pomegranate soft X-ray images. (**a**) Original image. (**b**) The image after threshold segmentation.

The threshold segmentation method was employed to separate pomegranate subjects from the background. By setting an appropriate grayscale threshold, image pixels were categorized into two groups: those with grayscale values exceeding the threshold and those below it. Through iterative experimental adjustments in this study, the optimal grayscale threshold was ultimately determined as 200. This process effectively isolated pomegranate specimens from the background, eliminating interference while highlighting structural information of the fruit subjects. The segmentation procedure is visually demonstrated in Figure 6.

The segmented images were subsequently subjected to noise reduction using a median filtering algorithm for soft X-ray images, followed by contrast enhancement through the Contrast-Limited Adaptive Histogram Equalization (CLAHE) algorithm [40,41]. As an advanced version of traditional histogram equalization, the CLAHE algorithm adaptively performs histogram equalization on different image regions, thereby mitigating the local over-enhancement issues inherent in conventional methods.

Texture features were extracted based on the gray-level co-occurrence matrix (GLCM) and the gray-gradient co-occurrence matrix (GGCM), generating 21 texture characteristic parameters whose computational formulas are detailed in Table 2 [42,43]. Among these, No.1–No.6 represent texture features derived from the GLCM, where angular second moment quantifies textural homogeneity and coarseness, correlation measures gray-level similarity along row or column directions, entropy indicates randomness in image information content, contrast reflects image clarity and textural depth, homogeneity describes textural fineness and complexity, while variance characterizes deviations in gray-level distribution. No.7–No.21 correspond to features extracted from the GGCM, which provide additional feature parameters for developing enhanced classification models.

Classification models for pomegranate blackheart disease were established by integrating these texture features with Random Forest (RF) and Support Vector Machine (SVM) algorithms [44,45]. Model performance was evaluated using accuracy rates and F1-scores from both training and testing sets as evaluation metrics. The entire preprocessing and modeling procedures were implemented using MATLAB 2023b (MathWorks, Natick, MA, USA) software.

## 3. Results and Discussion

### 3.1. Destructive Testing Results of Pomegranate Disease Severity

**Figure 7:**
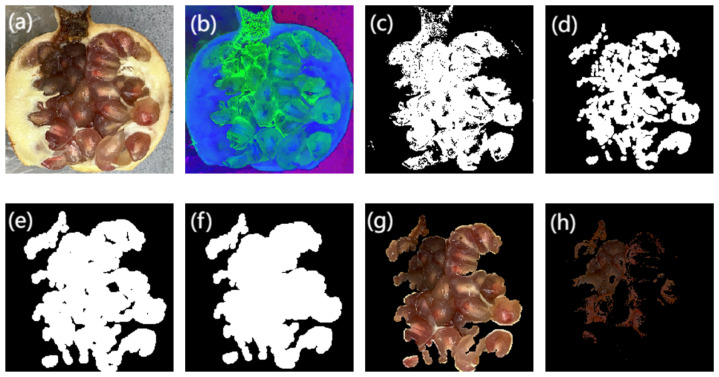
Image processing workflow for pomegranate cross-sections: (**a**) original RGB cross-sectional image (**b**) HSV color space conversion; (**c**) seed region extraction; (**d**–**f**) morphological operations; (**g**) seed segmentation; and (**h**) pathological region segmentation.

The internal tissue coloration of pomegranates is more complex compared to fruits like apples. To extract infected regions from cross-sectional images, this study converts RGB images of pomegranate sections into the HSV color space. The seed-containing regions within the pomegranate are segmented, and morphological operations are applied to remove background noise. Subsequently, the complete aril regions are reconstructed through iterative morphological dilation operations. Guided by expert evaluations, areas with brightness values below a predefined threshold are identified as blackheart disease regions. The pixel ratio of these diseased areas to the entire cross-sectional area is calculated to serve as a quantitative representation of the true infection severity. The image processing workflow for pomegranate cross-sections is illustrated in Figure 7.

As an internal pathological condition, blackheart disease has traditionally been evaluated using qualitative assessment methods. To enhance the discrimination of infection severity, this study employed destructive detection methods combined with image processing techniques to quantify the actual infection extent, which was subsequently classified into distinct severity grades. These categorized grades served as the output parameters for the discrimination models of pomegranate blackheart disease established through NIR spectroscopy/soft X-ray imaging technologies. The specific classification criteria are detailed in Table 3. Based on the dual classification of the area ratio of internal disease regions detected through destructive testing and the number of days after inoculation, this approach maintains an equal number of pomegranate samples for each disease severity category.

### 3.2. Descriptive Analysis of Healthy and Infected Pomegranate Samples

**Figure 8:**
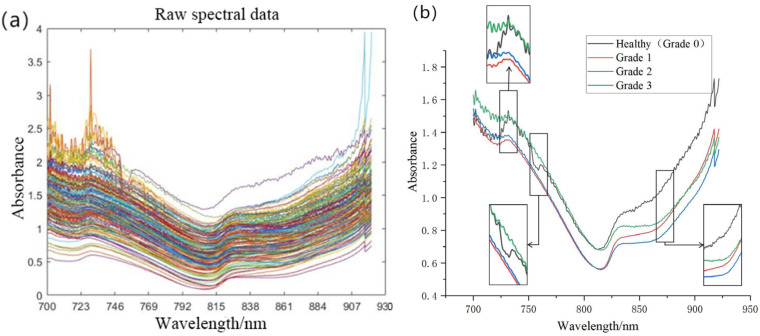
Spectral profiles of pomegranate specimens. (**a**) Absorbance curves of all specimens; (**b**) mean absorbance curves across infection severity grades.

Figure 8 displays both raw and mean spectral absorbance curves from all 192 pomegranate specimens spanning diverse infection severity grades. Although the overall spectral trends between healthy and infected specimens demonstrated similarity, statistically significant differences in absorbance were observed within specific wavelength ranges. Notably, healthy specimens exhibited distinct absorption peaks within both 725–750 nm and 750–775 nm spectral regions. In the 850–875 nm range, healthy samples manifested the most rapid rate of absorbance variation, whereas this rate progressively decreased with escalating infection severity, reaching its most attenuated state in Grade 3 specimens. Based on these descriptive observations, we hypothesize that derivative preprocessing algorithms may exert beneficial impacts on subsequent modeling analyses.

**Figure 9:**
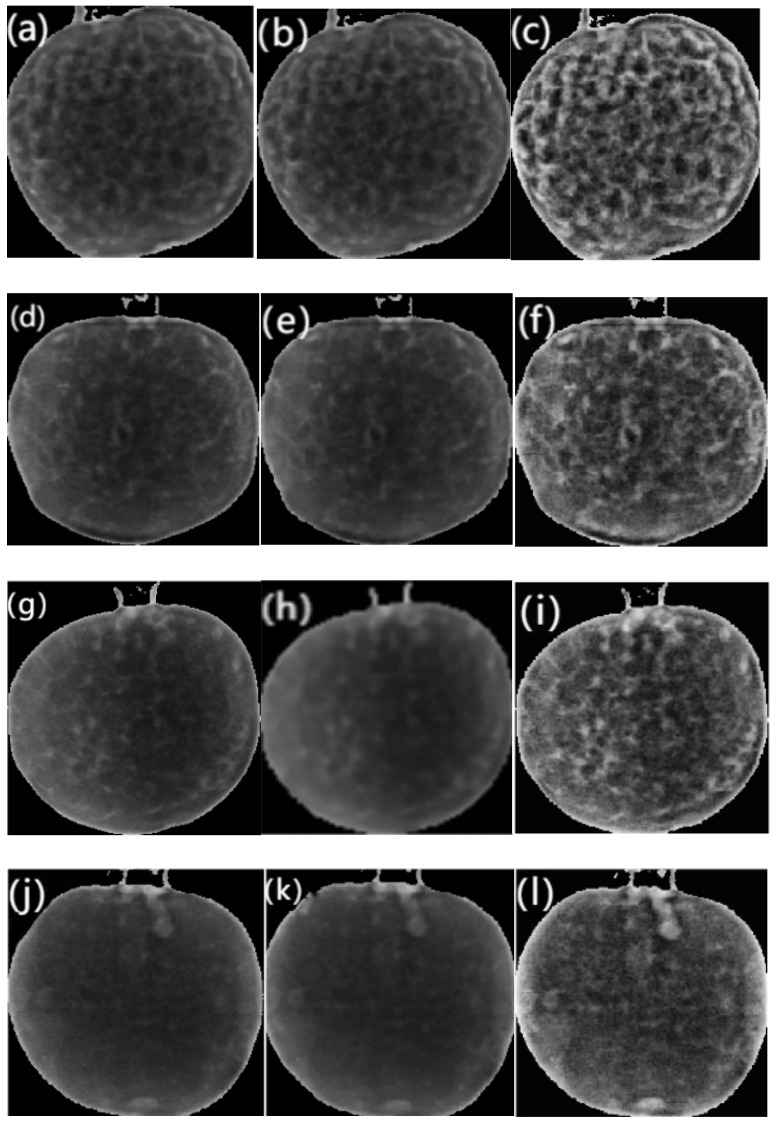
Soft X-ray image preprocessing (original image, after median filtering and CLAHE), (**a**–**c**) healthy specimens, (**d**–**f**) Grade 1 infected specimens, (**g**–**i**) Grade 2 infected specimens, and (**j**–**l**) Grade 3 infected specimens.

As shown in Figure 9, the soft X-ray images of pomegranate samples underwent preprocessing with median filtering and Contrast-Limited Adaptive Histogram Equalization (CLAHE). The postprocessed images exhibit significantly enhanced textural distinctions. Visual inspection of soft X-ray images across sample groups reveals that healthy pomegranates display tightly packed arils with uniform seed distribution in the images, attributable to the consistent density and structural integrity of uninfected tissues. In contrast, blackheart disease-infected samples exhibit localized hypodense regions or dark patches in the images, corresponding to fungal colonization sites where the cellular architecture undergoes progressive degradation. This pathological progression leads to seed dehydration and structural collapse, manifesting as reduced grayscale values and diminished textural complexity. However, such qualitative assessments remain inherently subjective and lack the capacity for precise quantitative staging of infection severity. Consequently, advanced computational approaches are required to extract objective, quantifiable features through systematic image analysis.

### 3.3. NIR Spectroscopy-Based Discriminant Model

#### 3.3.1. Outlier Elimination

Following standardized NIR spectroscopy preprocessing protocols, spectral outliers were systematically eliminated as the initial step to ensure data integrity. Both the Local Outlier Factor (LOF) algorithm and the Mahalanobis distance method employ threshold-based elimination of data points exhibiting significant deviations. The removal of outliers can effectively mitigate the impact of human-induced or random errors, thus enhancing data quality.

**Figure 10:**
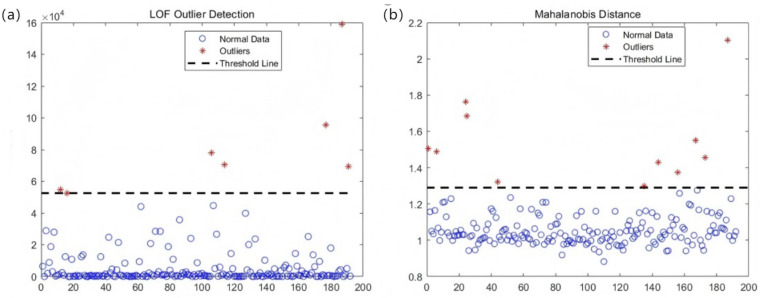
Removal of outliers, (**a**) LOF algorithm, (**b**) marginal distance algorithm.

As illustrated in Figure 10, the LOF algorithm retained 185 data points after removing 192 outliers, whereas the Mahalanobis distance method retained 181 data points. Notably, the anomalies detected by both methods predominantly originated from healthy pomegranate samples. This phenomenon may arise from class imbalance, where healthy samples numerically dominate other categories. In such cases, the majority class (healthy samples) typically exhibits a wider distribution within the data matrix, resulting in sparser inter-point distances that are computationally predisposed to outlier identification.

The performance metrics of the partial least squares discriminant analysis (PLS-DA) models constructed using the datasets remaining after applying two distinct outlier elimination algorithms are summarized in Table 3. As indicated in Table 4, the PLS-DA model derived from Mahalanobis distance-filtered data demonstrates superior performance compared to that based on Local Outlier Factor (LOF)-processed data. Specifically, the Mahalanobis distance approach yields higher classification accuracy on the test set, improved five-fold cross-validation mean accuracy, and enhanced F1-scores relative to the LOF method. However, a marginally lower F1-score observed in the test set may arise from stochastic variations in test set partitioning during simulation. Consequently, the Mahalanobis distance-filtered dataset was selected for subsequent blackheart disease detection trials in pomegranates due to its robust modeling performance.

#### 3.3.2. Spectral Denoising

**Figure 11:**
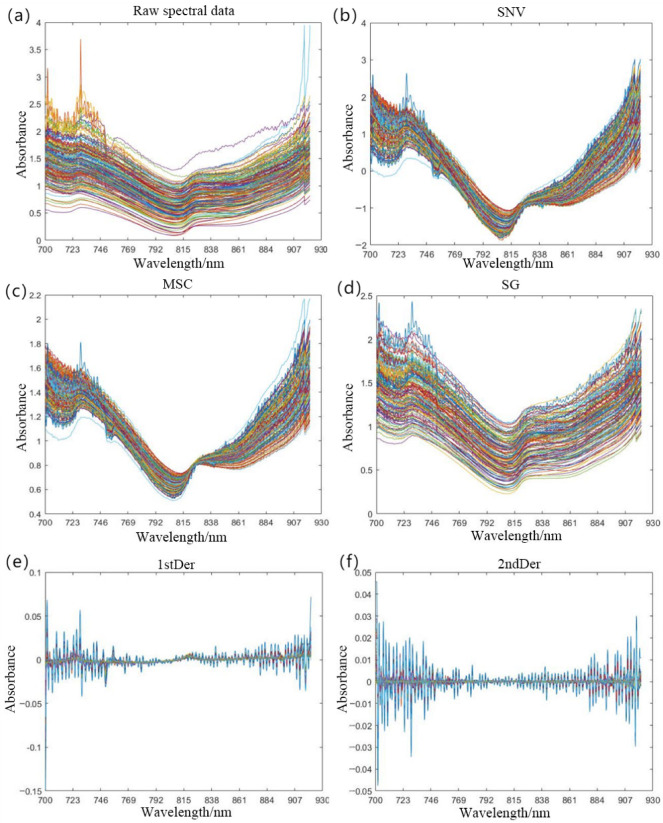
The spectral denoising processing results of pomegranate samples. (**a**) Raw spectral data. (**b**) Spectral preprocessed by the SNV algorithm. (**c**) Spectral preprocessed by the MSC algorithm. (**d**) Spectral preprocessed by the SG algorithm. (**e**) Spectral preprocessed by the D1 algorithm. (**f**) Spectral preprocessed by the D2 algorithm.

According to the processing results of different denoising algorithms shown in Figure 11, it can be observed that smoothing-based algorithms such as SNV, SG, and MSC can regularize the variation trends of spectral curves. The SNV algorithm effectively mitigates inter-sample physical variations; the SG smoothing algorithm eliminates high-frequency noise while preserving spectral trends; and the MSC algorithm standardizes spectral baselines.

However, this smoothing effect may potentially weaken characteristic features in specific spectral regions, thereby affecting modeling accuracy. Furthermore, the D1 (first derivative) algorithm accentuates absorption peak positions, and the D2 (second derivative) algorithm more effectively suppresses baseline drift.

To evaluate the efficacy of various denoising algorithms, we concurrently developed discrimination models for blackheart disease using both RF and SVM approaches. The denoised spectral data served as model inputs, whereas the outputs corresponded to pomegranate infection severity grades. The performance metrics of these algorithms are comprehensively summarized in Table 5.

As shown in Table 5, the discrimination model combining the second-derivative (D2) algorithm with the RF algorithm achieved optimal performance, with a prediction set accuracy of 84%. The choice of preprocessing methods significantly influenced the performance of RF in the discrimination model. In contrast, the D2 algorithm exhibited suboptimal performance in the SVM model, yielding a prediction set accuracy of only 72%. This phenomenon can be attributed to the derivative algorithm potentially enhancing the nonlinear structural relationships between features, thereby facilitating more effective data partitioning by RF. Conversely, SVM’s performance is highly dependent on sensitivity to feature scaling and reliance on high-dimensional information, leading to limited effectiveness under the same preprocessing condition. Concurrently, we observed a generalized performance degradation in SVM models constructed from denoised preprocessed data. This phenomenon was attributed to shifts in the optimal hyperparameter space induced by preprocessing operations, thereby necessitating further refinement of SVM hyperparameter tuning protocols.

Experimental results indicated that some predictions achieved an F1-score of 100% yet suboptimal accuracy, potentially due to class imbalance influencing model behavior. When data classes were imbalanced, the model tended to prioritize majority-class predictions. For instance, perfect precision and recall (both 100%) for healthy pomegranate samples would yield an F1-score of 100%, whereas misclassifications in blackheart disease samples across different infection stages led to reduced overall accuracy. To mitigate this bias, the number of healthy samples was adjusted from 96 to 32, matching the 32 blackheart disease samples (categorized by infection duration), thereby ensuring balanced datasets and enhancing experimental reliability.

#### 3.3.3. Feature Wavelength Selection

Following the adjustment of healthy sample size, spectral data were subjected to wavelength selection using CARS, SPA, and PCA algorithms, yielding 62, 50, and 8 feature wavelengths, respectively. Training and test sets were partitioned in a 7:3 ratio, and RF algorithm-based classification models for pomegranate blackheart disease were subsequently established. The performance metrics of these models are summarized in Table 6.

**Figure 12:**
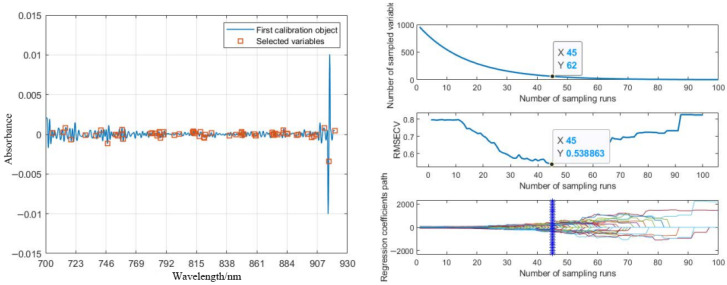
Process of optimal wavelength selection based on the CARS algorithm.

Figure 12 illustrates the feature wavelength selection process of the CARS algorithm. The analysis suggests that the CARS algorithm, through iterative adaptive reweighting, prioritizes stable and influential spectral features, thereby exhibiting enhanced noise resistance. Its adaptive mechanism effectively mitigates the impact of data noise, contributing to robust performance. In contrast, the SPA showed higher sensitivity to spectral noise, leading to suboptimal projection calculations due to interference from noisy signals. Meanwhile, PCA dimensionality reduction resulted in insufficient interpretability of original spectral features, limiting the classification accuracy of the model.

**Figure 13:**
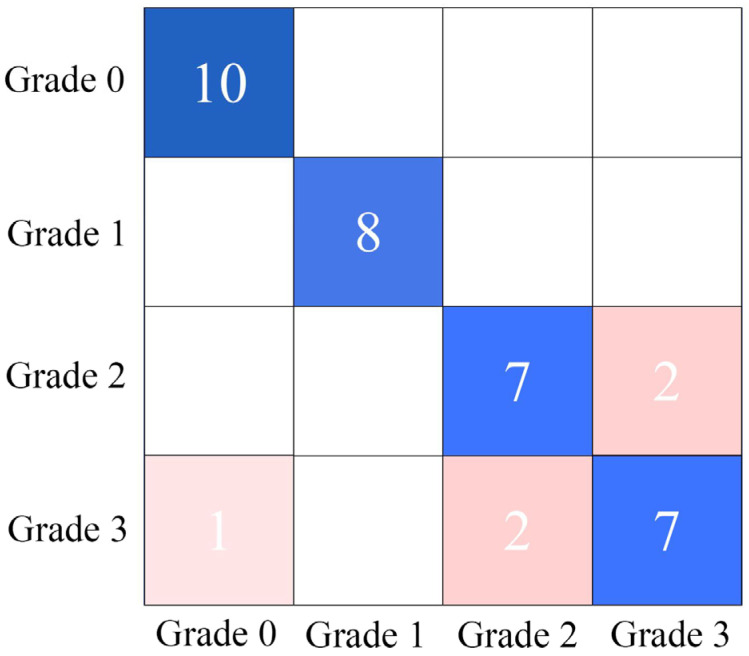
Confusion matrix of the prediction set for the optimal discrimination model of pomegranate blackheart disease established using NIR technology.

As shown in Table 6, the RF model incorporating CARS-based feature selection demonstrated superior performance, achieving a test set accuracy of 86.00%. Analysis of the confusion matrix results from the D2-CARS-RF model in Figure 13 revealed that misclassifications in the test set predominantly occurred between samples infected for Grade 2 and Grade 3. This is likely due to minimal differences in disease severity between these two stages, resulting in high data similarity that compromised the model’s classification performance.

### 3.4. Soft X-Ray Imaging-Based Discriminant Model

Using the computational formulas provided in Table 2 and the preprocessed images of all samples, the textural features of all samples were calculated. The mean values of each textural feature across different sample groups are presented in Table 7.

Using the textural feature data of all samples as the input and the infection severity grades of pomegranates as the output, RF and SVM classification models were constructed. The dataset was partitioned into training and test sets at a 7:3 ratio, with the training set utilized for model training and hyperparameter optimization and the test set reserved for evaluating model performance.

Through systematic experimentation, the hyperparameters of both RF and SVM models were optimized to identify optimal configurations for the experimental data. For the RF model the number of decision trees was set to 100 and the number of randomly selected features (mtry) was fixed at two. For the SVM model, comparative trials with different kernel functions (linear, polynomial, and radial basis function (RBF)) revealed that the RBF kernel achieved superior performance on this dataset. The optimal hyperparameters for SVM were determined as regularization parameter *C* = 10 and kernel coefficient *γ* = 0.1, which collectively enhanced classification accuracy.

**Figure 14:**
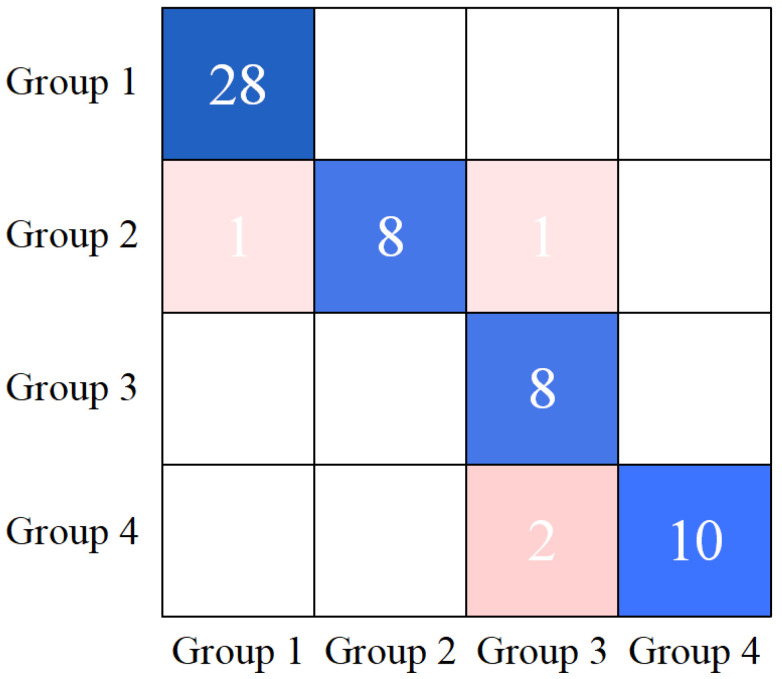
Confusion matrix of the prediction set for the optimal discrimination model of pomegranate blackheart disease established using soft X-ray imaging technology.

To validate the models’ capability to generalize to unseen samples, the performance of both the RF and SVM models was rigorously evaluated on the test set. The key performance metrics are summarized in Table 8. As shown in Table 8, the RF model achieved the highest performance on the test set, with an overall accuracy of 93.10%. The confusion matrix of the RF model is shown in Figure 14. Analysis of the confusion matrix revealed that the model achieved perfect classification accuracy (100%) for Grade 0 and Grade 2 samples. However, predictions for Grade 1 and Grade 3 samples each exhibited two misclassifications. The model demonstrated robust performance in identifying healthy pomegranates and those with Grade 2 blackheart disease infections, whilst showing minor inaccuracies in classifying Grade 1 and Grade 3 infected samples. Nevertheless, when evaluated through comprehensive metrics (overall accuracy and F1-score), the RF model exhibited strong classification capability. These findings collectively indicate that the RF model effectively discriminates pomegranate samples at different stages of blackheart disease progression.

### 3.5. NIR Spectroscopy vs. Soft X-Ray Imaging

Based on the experimental findings of this study, both detection technologies successfully discriminated pomegranate blackheart disease across varying infection severities, providing two novel approaches for pathological detection. The results demonstrated that the RF model exhibited optimal performance when combining either technique. Among the top-performing models, the discrimination model based on soft X-ray imaging achieved superior efficacy, attaining 93.10% prediction set accuracy and a 98.11% F1-score.

However, model performance was influenced by multiple factors including hardware specifications, sample size, and algorithm compatibility. Consequently, future research optimizations should focus on employing higher-precision spectrometers and soft X-ray imaging equipment, expanding the experimental sample size for modeling, and integrating advanced deep learning algorithms. Current detection methods rely solely on off-the-shelf commercial devices. Future adoption of custom-engineered spectrometers or soft X-ray imaging systems coupled with in-house developed software could substantially reduce ancillary costs and enhance the cost-effectiveness of detection methodologies.

Both detection techniques possess distinct advantages. NIR spectroscopy fundamentally reflects the internal chemical properties of pomegranates by detecting the response of hydrogen-containing groups to light at different wavelengths. During transmission-mode detection, future studies could combine chemometric methods to simultaneously evaluate physicochemical indices (e.g., pH, soluble solid content, and titratable acidity) while diagnosing blackheart disease. Soft X-ray imaging primarily identifies internal physical characteristics by detecting density variations across different regions of pomegranates. With the integration of conveyor belts and high-precision imaging devices, this technique holds potential for continuous high-throughput detection of large sample batches. Simultaneously, future research could employ information fusion techniques to construct discriminative models by integrating data from dual sensors, thereby further enhancing model accuracy.

## 4. Conclusions

In this study, pomegranate samples with blackheart disease at different infection stages were prepared through artificial fungal inoculation. Transmission-mode NIR spectroscopy was employed to collect spectral data from healthy and infected samples, while soft X-ray imaging was simultaneously applied to acquire internal structural images. Visual assessment of average absorbance differences across infection stages revealed significant spectral variations in the 725–775 nm and 850–875 nm wavelength ranges. Comparative analysis of soft X-ray images demonstrated that healthy pomegranates exhibited distinct seed texture features, which progressively diminished with increasing disease severity.

Blackheart disease prediction models were developed based on NIR spectral data and preprocessing methods, incorporating five denoising algorithms. The model utilizing second-derivative preprocessing demonstrated the best predictive performance, achieving a prediction set accuracy of 84.00% and an F1-score of 98.11%. Further optimization through three feature wavelength selection algorithms identified the D2-CARS-RF model as optimal with 62 selected wavelengths, attaining a prediction set accuracy of 86.00% and an F1-score of 89.33%.

For soft X-ray image analysis, preprocessing techniques including median filtering and CLAHE were implemented. Twenty-one textural features were extracted from all samples using GLCM and GGCM methods to establish classification models. The RF model exhibited superior performance, achieving a prediction set accuracy of 93.10% and an F1-score of 98.11%. The established model demonstrated robust performance in discriminating pomegranate blackheart disease across varying infection severities.

This study proposes two novel methodologies for blackheart disease discrimination, validating NIR spectroscopy and soft X-ray imaging as valuable tools for assessing disease progression. Technical strengths and limitations were systematically analyzed based on experimental outcomes. Industrial implementation of these techniques could enhance consumer safety, protect commercial interests, and promote sustainable development in the pomegranate industry.

## Figures and Tables

**Table 1:** Fungal inoculation experimental protocol for pomegranates.

Fungal Inoculation Time Point	Harvest Time Point	Infection Time	Quantity	Storage Conditions
/	/	0 day	96	Ambient temperature and humidity
Day 3	Day 4	1 day	32	20 °C, 90% RH
Day 2	Day 4	2 days	32	20 °C, 90% RH
Day 1	Day 4	3 days	32	20 °C, 90% RH

**Table 2:** Image texture feature calculation formula.

No.	Texture Feature	Formula
1	Angular Second Moment	$\sum_{i}\sum_{j}{P(i,j)}^{2}$
2	Correlation	$\sum_{i}\sum_{j}((ij)P(i,j)-\mu_{x}\mu_{y})/\sigma_{x}\sigma_{y}$
3	Entropy	$-\sum_{i}\sum_{j}P(i,j)\log p(i,j)$
4	Contrast	$\sum_{i}\sum_{j}{\left(i-j\right)}^{2}P(i,j)$
5	Homogeneity	$\sum_{i}\sum_{j}P(i,j)/(1+\left|i-j\right|)$
6	Variance	$\sum_{i}\sum_{j}P\left(i,j\right)\;*\;{\left(i-\sum_{i}\sum_{j}P\left(i,j\right)\;*\;i\right)}^{2}$
7	Small Gradient Advantage	$T_{1}=\left[\sum_{i}\sum_{j}\frac{H\left(i,j\right)}{j^{2}}\right]/\left[\sum_{i}\sum_{j}H\left(i,j\right)\right]$
8	Large Gradient Advantage	$T_{2}=\left[\sum_{i}\sum_{j}j^{2}H\left(i,j\right)\right]/\left[\sum_{i}\sum_{j}H\left(i,j\right)\right]$
9	Gray Non-uniformity	$T_{3}=\left\{\sum_{i}{\left[\sum_{j}H\left(i,j\right)\right]}^{2}\right\}/\left[\sum_{i}\sum_{j}H\left(i,j\right)\right]$
10	Gradient Non-uniformity	$T_{4}=\left\{\sum_{j}{\left[\sum_{i}H\left(i,j\right)\right]}^{2}\right\}/\left[\sum_{i}\sum_{j}H\left(i,j\right)\right]$
11	Energy	$E=\sum_{i}\sum_{j}{P\left(i,j\right)}^{2}$
12	Gray Mean	$\mu_{1}=\sum_{i}i\left[\sum_{j}P\left(i,j\right)\right]$
13	Gradient Mean	$\mu_{2}=\sum_{j}j\left[\sum_{i}P\left(i,j\right)\right]$
14	Gray Variance	$\partial_{1}=\sqrt{\left\{\sum_{i}{\left(i-\mu_{1}\right)}^{2}\left[\sum_{j}P\left(i,j\right)\right]\right\}}$
15	Gradient Variance	$\partial_{2}=\sqrt{\left\{\sum_{j}{\left(j-\mu_{2}\right)}^{2}\left[\sum_{i}P\left(i,j\right)\right]\right\}}$
16	Correlation	$T_{5}=\frac{1}{\partial_{1}\partial_{2}}\sum_{i}\sum_{j}\left(i-\mu_{1}\right)\left({j-\mu }_{2}\right)P\left(i,j\right)$
17	Gray Entropy	$T_{6}=-\left\{\sum_{i}\left[\sum_{j}P\left(i,j\right)\right]\log\left[\sum_{j}P\left(i,j\right)\right]\right\}$
18	Gradient Entropy	$T_{7}=-\left\{\sum_{j}\left[\sum_{i}P\left(i,j\right)\right]\log\left[\sum_{i}P\left(i,j\right)\right]\right\}$
19	Hybrid Entropy	$T_{8}=-\sum_{i}\sum_{j}P\left(i,j\right)\log P\left(i,j\right)$
20	Inertia	$T_{9}=\sum_{i}\sum_{j}{\left(i,j\right)}^{2}P\left(i,j\right)$
21	Inverse Difference Moment	$T_{10}=\sum_{i}\sum_{j}P\left(i,j\right)/(1+{\left(i-j\right)}^{2})$

$\mu_{x}$—row mean of the gray-level co-occurrence matrix; $\mu_{y}$—column mean of the gray-level co-occurrence matrix; $\sigma_{x}$—row standard deviation of the gray-level co-occurrence matrix; $\sigma_{y}$—column standard deviation of the gray-level co-occurrence matrix; *P*(*i*,*j*)—element value at position (*i*,*j*) of the gray-level co-occurrence matrix; *H*(*i*,*j*)—element value at position (*i*,*j*) of the gray-level gradient co-occurrence matrix.

**Table 3:** Statistical results of disease severity in pomegranate samples.

Disease Severity (Grade)	Infection Time	Disease Severity (%)	Quantity
0	0 day	0	96
1	1 day	3.2–7.8	32
2	2 days	7.9–18.6	32
3	3 days	18.7–30.4	32

**Table 4:** Performance of PLS-DA models constructed using data remaining after applying two outlier elimination algorithms.

Algorithms	Test Set Accuracy	F1-Score	Five-Fold Cross-Validation Mean Accuracy	Five-Fold Cross- Validation Mean F1-Score	Number of Correct Classifications	Number of Incorrect Classifications
LOF	52.11%	45.33%	66.71%	65.38%	95	86
Mahalanobis distance	54.54%	44.91%	73.72%	67.11%	99	82

**Table 5:** Performance metrics of the pomegranate blackheart disease discrimination models established based on raw spectra and five denoising algorithms.

Models	Training Set Accuracy	Training Set F1-Score	Test Set Accuracy	Test Set F1-Score
RF	87.17%	93.75%	56.00%	78.57%
SNV-RF	94.01%	95.72%	56.00%	77.41%
MSC-RF	93.16%	96.72%	68.00%	89.65%
SG-RF	83.76%	92.53%	62.00%	83.33%
D1-RF	99.14%	100.0%	82.00%	100.0%
D2-RF	99.14%	100.0%	84.00%	98.11%
SVM	89.74%	100.0%	80.00%	96.29%
SNV-SVM	78.63%	98.30%	74.00%	98.24%
MSC-SVM	79.48%	97.71%	70.00%	87.50%
SG-SVM	88.03%	100.0%	82.00%	100.0%
D1-SVM	89.74%	98.38%	66.00%	100.0%
D2-SVM	90.50%	99.13%	72.00%	90.90%

**Table 6:** Performance metrics of the pomegranate blackheart disease discrimination models established based on full-wavelength spectra and three feature selection algorithms.

Models	Wavelength Number	Training Set Accuracy	Training Set F1-Score	Test Set Accuracy	Test Set F1-Score
D2-RF	958	99.14%	100.0%	84.00%	98.11%
D2-CARS-RF	62	97.43%	99.13%	86.00%	89.33%
D2-SPA-RF	50	95.21%	98.26%	80.00%	83.33%
D2-PCA-RF	8	79.48%	77.71%	74.00%	78.24%

**Table 7:** Mean values of textural features across infection severity grades.

No.	Texture Feature	Grade 0 (Healthy)	Grade 1	Grade 2	Grade 3
1	Angular second Moment	0.3862	0.4371	0.3998	0.4066
2	Correlation	−33.20	−23.31	−32.92	−30.41
3	Entropy	7.151	6.732	6.969	6.695
4	Contrast	2.913 × 10^6^	2.542 × 10^6^	2.791 × 10^6^	2.526 × 10^6^
5	Homogeneity	1.707	1.745	1.721	1.745
6	Variance	6.158	5.764	6.066	5.778
7	Small gradient Advantage	0.7523	0.7217	0.7694	0.8144
8	Large gradient Advantage	0.6349	0.6978	0.6011	0.5316
9	Gray non-uniformity	4742	5231	4830	4405
10	Gradient Non-uniformity	3.476 × 10^4^	3.241 × 10^4^	3.625 × 10^4^	4.035 × 10^4^
11	Energy	0.0663	0.0733	0.0676	0.0616
12	Gray mean	48.72	43.48	48.33	45.43
13	Gradient mean	0.6349	0.5779	0.6011	0.5316
14	Gray variance	37.62	36.03	37.14	34.45
15	Gradient variance	2.176	2.072	2.174	2.106
16	Correlation	22.42	22.46	20.94	19.34
17	Gray entropy	1.718	1.666	1.702	1.692
18	Gradient entropy	0.3972	0.3372	0.3787	0.3451
19	Hybrid entropy	1.989	1.896	1.962	1.929
20	Inertia	3705	3093	3629	3178
21	Inverse difference moment	0.2483	0.2694	0.2504	0.2349

**Table 8:** Performance metrics of the pomegranate blackheart disease discrimination model established based on texture feature parameters.

Models	Grade	Number of Samples	Number of Errors	Accuracy	F1-Score
RF	0	28	0	93.10%	98.11%
	1	10	2		
	2	8	0		
	3	12	2		
SVM	0	31	0	81.03%	67.91%
	1	11	11		
	2	7	0		
	3	9	0		

## Data Availability

The original contributions presented in the study are included in the article, further inquiries can be directed to the corresponding author.
